# The nature of tremor circuits in parkinsonian and essential tremor

**DOI:** 10.1093/brain/awu250

**Published:** 2014-09-08

**Authors:** Hayriye Cagnan, Simon Little, Thomas Foltynie, Patricia Limousin, Ludvic Zrinzo, Marwan Hariz, Binith Cheeran, James Fitzgerald, Alexander L. Green, Tipu Aziz, Peter Brown

**Affiliations:** 1 Nuffield Department of Clinical Neurosciences, University of Oxford, John Radcliffe Hospital, West Wing Level 6, OX3 9DU, Oxford, UK; 2 Sobell Department of Motor Neuroscience and Movement Disorders, UCL Institute of Neurology, London, WC1N 3BG, UK; 3 Nuffield Department of Surgical Sciences, John Radcliffe Hospital, University of Oxford, Oxford, OX3 9DU, UK

**Keywords:** basal ganglia, deep brain stimulation, tremor, clinical neurophysiology, thalamus

## Abstract

Tremor is a cardinal feature of Parkinson’s disease and essential tremor, the two most common movement disorders. Yet, the mechanisms underlying tremor generation remain largely unknown. We hypothesized that driving deep brain stimulation electrodes at a frequency closely matching the patient’s own tremor frequency should interact with neural activity responsible for tremor, and that the effect of stimulation on tremor should reveal the role of different deep brain stimulation targets in tremor generation. Moreover, tremor responses to stimulation might reveal pathophysiological differences between parkinsonian and essential tremor circuits. Accordingly, we stimulated 15 patients with Parkinson’s disease with either thalamic or subthalamic electrodes (13 male and two female patients, age: 50–77 years) and 10 patients with essential tremor with thalamic electrodes (nine male and one female patients, age: 34–74 years). Stimulation at near-to tremor frequency entrained tremor in all three patient groups (ventrolateral thalamic stimulation in Parkinson’s disease, *P* = 0.0078, subthalamic stimulation in Parkinson’s disease, *P* = 0.0312; ventrolateral thalamic stimulation in essential tremor, *P* = 0.0137; two-tailed paired Wilcoxon signed-rank tests). However, only ventrolateral thalamic stimulation in essential tremor modulated postural tremor amplitude according to the timing of stimulation pulses with respect to the tremor cycle (e.g. *P* = 0.0002 for tremor amplification, two-tailed Wilcoxon rank sum test). Parkinsonian rest and essential postural tremor severity (i.e. tremor amplitude) differed in their relative tolerance to spontaneous changes in tremor frequency when stimulation was not applied. Specifically, the amplitude of parkinsonian rest tremor remained unchanged despite spontaneous changes in tremor frequency, whereas that of essential postural tremor reduced when tremor frequency departed from median values. Based on these results we conclude that parkinsonian rest tremor is driven by a neural network, which includes the subthalamic nucleus and ventrolateral thalamus and has broad frequency-amplitude tolerance. We propose that it is this tolerance to changes in tremor frequency that dictates that parkinsonian rest tremor may be significantly entrained by low frequency stimulation without stimulation timing-dependent amplitude modulation. In contrast, the circuit influenced by low frequency thalamic stimulation in essential tremor has a narrower frequency-amplitude tolerance so that tremor entrainment through extrinsic driving is necessarily accompanied by amplitude modulation. Such differences in parkinsonian rest and essential tremor will be important in selecting future strategies for closed loop deep brain stimulation for tremor control.

## Abstract (precis)

See Arkadir *et al.* (doi:10.1093/brain/awu285) for a scientific commentary on this article. The mechanisms underlying tremor generation remain unclear. Cagnan *et al.* use deep brain stimulation of the thalamus or subthalamic nucleus at/near a patient's own tremor frequency to investigate the networks responsible for parkinsonian and essential tremor. The results reveal differences in the circuitry underlying these two tremor types.

## Introduction

Tremor, as most commonly seen in Parkinson’s disease and essential tremor, is associated with brain activity at tremor frequency or double this. During essential tremor, thalamic neurons exhibit firing patterns correlated with tremor, predominantly in the cerebellar input receiving zone, the ventralis intermedius (Hua and Lenz, 2005). In Parkinson’s disease, tremor-related neural activity has been demonstrated both in the cerebello-thalamo-cortical circuit, and in the basal ganglia and related receiving areas of the thalamus (Lenz *et al.*, 1994; Bergman *et al.*, 1998; Hurtado *et al.*, 1999; Magnin *et al.*, 2000; Rodriguez-Oroz *et al.*, 2001; Timmermann *et al.*, 2002; Reck *et al.*, 2009; Hirschmann *et al.*, 2013).

However, in what capacity are the basal ganglia and cerebellar systems involved in the generation of parkinsonian tremor? The fact that tremor may be suppressed by high frequency stimulation, or indeed lesioning, at these sites (Benabid *et al.*, 1991; Lenz *et al.*, 1995; Krack *et al.*, 1998; Fasano *et al.*, 2012) need not necessarily imply interference with a pacemaker circuit, and could potentially arise from interference with the pacemaker circuit’s outflow or mechanisms amplifying this outflow. Consider, for example, the effect of disrupting cortical outflow by either extirpating the motor cortex or sectioning the corticospinal tracts. These procedures abolished tremor but did not necessarily accomplish this by annihilating central tremor oscillations, only their peripheral consequences through disruption of the final common motor pathway (Oliver, 1949). One way to demonstrate that a site is involved in the tremor oscillation-generating pacemaker circuit (including its inputs), rather than in propagating already established oscillations to anterior horn cells, is to establish whether low frequency stimulation can entrain tremor through modulation of the oscillatory neural activity at the stimulation site (Walker *et al.*, 1982; Ermentrout, 1996; Smeal *et al.*, 2010; Cagnan *et al.*, 2013). Low frequency stimulation of pathways that propagate established oscillations would not lead to entrainment of the latter. However, central sites may also be important in dictating the amplitude of tremor. Tremor entrainment and amplitude modulation can potentially be separated, as suggested in recent imaging studies in patients with Parkinson’s disease (Helmich *et al.*, 2011), or combined (Cagnan *et al.*, 2013). The distinction between these different functions is potentially important, as the involvement of a given structure in a pacemaker circuit opens up the possibility of targeting specific instances in the cycle (i.e. phases) of pathological neural oscillations to more efficiently promote clinically significant tremor control (Tass and Majtanik, 2006; Brittain *et al.*, 2013; Cagnan *et al.*, 2013).

In this study, we investigate the role of the ventrolateral thalamus and the subthalamic nucleus in parkinsonian rest tremor by driving these nuclei through deep brain stimulation (DBS) at near tremor frequencies and assessing the degree of entrainment and amplitude modulation of tremor. We compare the response of parkinsonian rest tremor to stimulation at near tremor frequencies to that observed in essential tremor patients during ventrolateral thalamic stimulation (Cagnan *et al.*, 2013). The findings point to fundamental differences in the nature of the underlying tremor network in the two conditions.

## Materials and methods

### Patients and recordings

All patients gave their informed consent to take part in this study, which was approved by the local research ethics committees of the University of Oxford and University College of London. We recorded from eight patients with Parkinson’s disease who had undergone unilateral or bilateral implantation of DBS electrodes into the ventrolateral thalamus, seven patients with Parkinson’s disease implanted bilaterally in the subthalamic nucleus (Table 1) and 10 patients with essential tremor implanted unilaterally or bilaterally in the ventrolateral thalamus (Table 2). Note that the tremor-dominant Parkinson’s disease patients implanted in the ventrolateral thalamus were poorly dopaminergic responsive [mean percentage drop in Unified Parkinson’s Disease Rating Scale (UPDRS) motor score when medication was ingested after overnight withdrawal was 16 ± 15% (SEM) one sample *t*-test, df = 5, *P* = 0.3271; mean percentage drop in UPDRS rest tremor score was 9.7 ± 6% one sample *t*-test, df = 5, *P* = 0.1801]. For those patients with Parkinson’s disease implanted in the subthalamic nucleus, mean percentage drop in UPDRS motor score when medication was ingested after overnight withdrawal was 68 ± 6% (one sample *t*-test, df = 6, *P* < 0.0001); whereas mean percentage drop in the UPDRS rest tremor score was 46 ± 12% (one sample *t*-test, df = 5, *P* = 0.0249). The patients described with essential tremor were those previously reported in another study (Cagnan *et al.*, 2013). However, the essential tremor data presented have been re-analysed so that they are treated in the same way as the recordings made in Parkinson’s disease, and further novel analyses are presented.

**Table 1:** Clinical details of patients with Parkinson’s disease with subthalamic or ventrolateral thalamic DBS electrodes

	Age	Gender	Most affected limb	Disease duration, years	Pre-op UPDRS OFF	Pre-op UPDRS ON	Electrode implantation (months)	Stimulation parameters
	Ventrolateral thalamus							
1	65	M	RH	6	29	N/A	24	3 - B + 3 V, 210 µs, 4 Hz
2	65	M	LH	7	35	20	27	1 - B + 2.6 V, 210 µs, 5 Hz
3	66	M	RH	9	24	31	14	0 - B + 4.0 V, 210 µs, 4 Hz
4	61	M	RH	5	21	6	0	0 - 3 + 2.5 V, 210 µs, 5 Hz
5	77	F	LH	6	45	39	0	0 - 3 + 1.5 V, 210 µs, 5 Hz
6	75	F	LH	11	29	34	18	2 - B + 2.2 V, 210 µs, 4 Hz
7	66	M	RH	11	N/A	N/A	0	0 - 3 + 3 V, 210 µs, 5 Hz
8	73	M	RH	7	39	32	13	0 - B + 2.8 V, 210 µs, 4 Hz
	Subthalamic nucleus							
9	57	M	RH	10	27	5	42	0 - B + 3.8 V, 210 µs, 5 Hz
10	60	M	RH	12	41	20	13	1 - B + 3.4 V, 210 µs, 4 Hz
11	50	M	RH	12	44	7	3	2 - B + 2.5 V, 210 µs, 5 Hz
12	64	M	LH	13	52	8	11	1 - B + 2.1 V, 210 µs, 4 Hz
13	52	M	LH	10	38	15	19	1 - B + 3.2 V, 210 µs, 4 Hz
14	72	M	RH	8	38	19	29	0 - B + 3.2 V, 210 µs, 4 Hz
15	59	M	RH	14	52	19	22	1 - B + 3.0 V, 210 µs, 4 Hz

RH = right hand; LH = left hand; B = battery when stimulation is grounded to the implanted pulse generator.

All Parkinson’s disease patients were stimulated with the same pulse width (210 µs), and there were no significant differences between stimulation frequencies or voltages (*P* = 0.7552 and *P* = 0.2946, respectively; two-tailed Wilcoxon rank sum tests) between the different surgical targets.

**Table 2:** Clinical details of patients with essential tremor with ventrolateral thalamic DBS electrodes

	Age	Gender	Most affected limb	Disease duration, years	Pre-op tremor score	Electrode implantation (months)	Stimulation parameters
16	59	M	RH	37	14	48	0 - B + 3.6 V, 210 µs, 5 Hz
17	70	M	LH	52	14	24	1 - B + 2.2 V, 240 µs, 7 Hz
18	67	M	LH	60	17	12	0-1 - B + 2.5 V, 210 µs, 4 Hz
19	55	M	LH	35	15	18	0 - B + 1.7 V, 210 µs, 5 Hz
20	71	F	RH	29	14	18	0 - B + 3.5 V, 210 µs, 4 Hz
21	73	M	RH	7	23	12	1 - B + 1.5 V, 240 µs, 6 Hz
22	61	M	LH	55	23	7	2 - B + 2.7 V, 210 µs, 6 Hz
23	56	M	RH	38	15	0	2 + 1 - 2.5 V, 210 µs, 5 Hz
24	74	M	RH	28	26	1	2 - B + 2.0 V, 210 µs, 4 Hz
25	34	M	RH	11	21	10	0 - B + 2.0 V, 210 µs, 6 Hz

RH = right hand; LH = left hand; B = battery when stimulation is grounded to the implanted pulse generator.

Preoperative tremor score is shown in the Bain and Findley scale (Bain *et al.*, 1993). All but one patient with essential tremor were stimulated with the same pulse width (210 µs), and there were no significant differences between stimulation frequencies or voltages between these patients and those with Parkinson’s disease implanted in the ventrolateral thalamus (*P* = 0.1883 and *P* = 0.3023, respectively; two tailed Wilcoxon rank sum tests).

Silver/silver chloride EEG electrodes were placed over Cz and Fz and a tri-axial accelerometer (Twente Medical Systems International) was attached to the index finger of the hand most affected by rest tremor for patients with Parkinson’s disease or postural tremor for patients with essential tremor. We opted to use accelerometry as our index of tremor for two principal reasons. First, we necessarily had to stimulate deep brain targets with a monopolar electrode configuration so as to detect stimulation timing from the stimulus artefact recorded from the scalp. However, the same artefact may contaminate EMG signals, making it more difficult to analyse these. Accelerometry was unaffected by stimulation artefact. Second, EMG is inevitably subject to a potential sampling bias. We could only have sampled from a proportion of the muscles in the hand and forearm, and so any amplitude and entrainment effects induced by stimulation may have been missed or, equally importantly, may have not been representative when detected in sampled EMG. By using a compound measure like accelerometry, we can at least be sure that any change in amplitude or entrainment upon stimulation relates to the bulk of muscle action. Furthermore, we have previously shown that accelerometry is sensitive to phase-dependent amplitude modulation of parkinsonian resting tremor during transcranial alternating current stimulation of the motor cortex despite a rather weaker entrainment effect than reported here (Brittain *et al.*, 2013). However, our policy of following tremor through accelerometry came at the expense of resolution, in that we were not able to dissociate which muscles were affected and if those affected varied across time. In addition, as considered further below, EMG recordings would have provided important insight to the relative contributions of central and peripheral mechanical effects on tremor resonance functions.

EEGs and the tri-axial accelerometer signal were recorded using a TMSI porti amplifier (Twente Medical Systems International), sampled at 2048 Hz and low-pass filtered at 500 Hz. Two recordings were made while subjects sat in a chair with their eyes open: (i) while DBS was switched off; and (ii) during unilateral stimulation delivered at the nearest integer frequency to the tremor frequency (*f_T_*) from the contralateral electrode to the most affected limb (Tables 1 and 2). Stimulation was delivered via the chronically implanted neurostimulator, which was programmed to parameters shown in Tables 1 and 2 using the N’vision telemetry control device (Medtronic Neurologic Division) in all patients apart from Patients 4, 5, 7 and 23. In these patients stimulation was controlled using the DualStim external stimulator (Medtronic Neurologic Division).

During the recordings acquired from patients with Parkinson’s disease, patients rested their hands on their lap in a pronated position throughout the two recording blocks. For analysis, we isolated time segments of minimum 5 s long, during which instantaneous tremor amplitude remained within the 2.5th and 97.5th percentiles of the overall tremor amplitude observed in that recording block. These segments were on average 17 ± 1 (SEM) seconds long across all patients and the two recording blocks. Recording segments were concatenated and treated as a continuous recording for each patient. In total 206 ± 15 s (SEM) of recording were analysed for each recording block. We only analysed data that lay within the 2.5th and 97.5th percentiles of overall tremor amplitude to reduce the amplitude variance in Parkinson’s disease, so that it more closely matched that in essential tremor. Reports are mixed within the literature as to whether tremor amplitude variance is greater in parkinsonian rest tremor than in essential tremor (Gao, 2004) or not (O’Suilleabhain and Matsumoto, 1998; Jankovic and Tolosa, 2007).

During the recordings acquired from patients with essential tremor, patients were asked to assume a tremor provoking posture. In six patients the tremor provoking upper limb posture entailed holding their most affected limb outstretched in front, with the wrist slightly extended (Patients 16, 17, 19, 20, 21 and 25). In four patients tremor was more marked with the shoulder abducted, elbow flexed and wrist extended (Patients 18, 22, 23 and 24). To minimize fatigue, postures were maintained for on average 75 ± 8 s (SEM), and followed by 30 s of rest before the arm was positioned again. We analysed tremor segments 2 s after posture was assumed to ensure stability of tremor recording. To match the length of analysis segments between the two patient cohorts, essential tremor recordings were divided into 17 s long segments. Recording segments were concatenated and treated as a continuous recording for each patient. On average 285 ± 14 s (SEM) of recording were analysed for each block (i.e. DBS at *f_T_* or turned off).

### Data analysis

EEGs and tri-axial accelerometer signals were analysed offline using MATLAB®. Tri-axial accelerometer signals were band-pass filtered forwards and backwards ± 2 Hz around the peak tremor frequency using a fourth order Butterworth filter. Peak tremor frequency was determined by visual inspection of the power spectral density estimate of the tri-axial accelerometer signal. Tremor amplitude envelope and instantaneous tremor phase were derived using the Hilbert Transform (Marple, 1999; Cagnan *et al.*, 2013). Tremor frequency was estimated by differentiating the unwrapped tremor phase followed by smoothing for 0.5 s. EEG signals were high-pass filtered using a fourth order Butterworth filter with a cut-off frequency of 100 Hz to derive the timing of each DBS pulse from the stimulation artefacts (Cagnan *et al.*, 2013).

The effect of stimulation on tremor was determined from the instantaneous phase and amplitude envelopes of the tri-axial accelerometer signals. The accelerometer axis, which showed the highest tremor entrainment during stimulation at *f_T_*, was used to represent tremor phase and instantaneous tremor amplitude for that patient. Instantaneous tremor phase and amplitude were either sampled at stimulation time points or at *f_T_* if DBS was switched off (Cagnan *et al.*, 2013). Instantaneous percentage change in tremor amplitude envelope was computed with respect to the median tremor amplitude of the corresponding recording segment. We opted to normalize instantaneous tremor amplitude with respect to the median amplitude within segments rather than with respect to the median tremor amplitude observed during an entire recording to minimize the effects of slow drifts in tremor amplitude.

### Tremor entrainment

**Figure 1:**
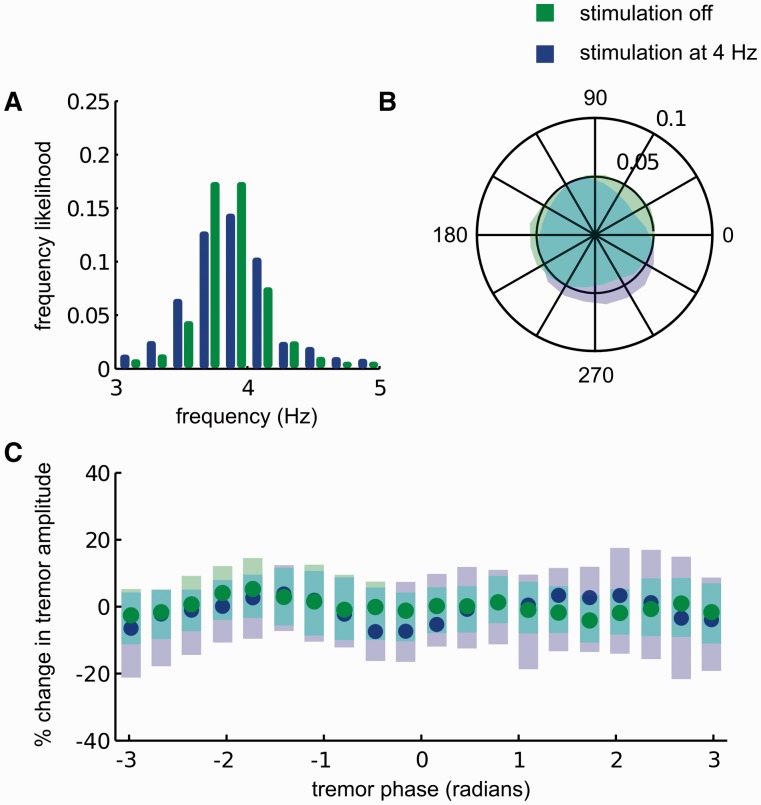
An exemplar of the effect of thalamic stimulation at *f_T_* in a patient with Parkinson’s disease. During stimulation at 4 Hz (**A**) median tremor frequency remained at 3.8 Hz, while (**B**) tremor phase during stimulation was pulled to the phase quadrant extending from 210 to 330° indicating significant tremor entrainment. Tremor phase when stimulation was switched off did not show any clear phase preference. Outer circle of the polar plot corresponds to tremor phase likelihood of 0.1, whereas the inner circle corresponds to tremor phase likelihood of 0.05. (**C**) During stimulation at *f_T_*, the instantaneous amplitude, derived from the tremor envelope, was not modulated depending on the timing of stimulation pulses with respect to the tremor cycle. Significance was assessed at each phase bin with respect to instantaneous tremor amplitude variability when stimulation was switched off using the Wilcoxon rank sum test and significance levels corrected for multiple comparisons using the false discovery rate procedure. Circles show median change in tremor amplitude and shaded regions indicate the 95th confidence intervals of the median values.

**Figure 2:**
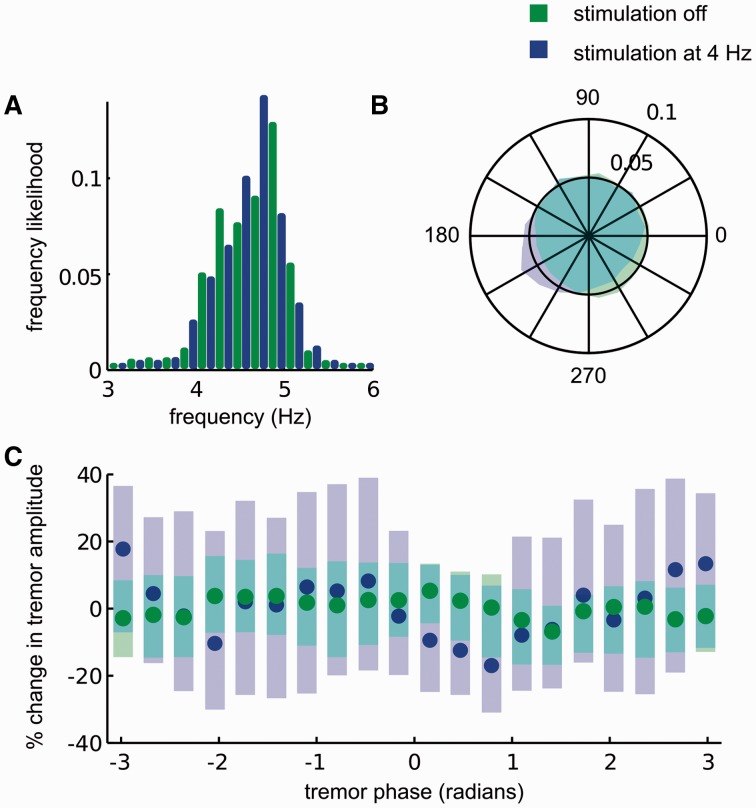
An exemplar effect of subthalamic stimulation at *f_T_* in a patient with Parkinson’s disease. (**A**) Median tremor frequency remained unchanged at 4.6 Hz during stimulation at 4 Hz. (**B**) Tremor phase was pulled to a region extending from 180 to 240° indicating significant tremor entrainment during stimulation. Tremor phase when stimulation was switched off was uniformly distributed around the unit circle. Outer circle corresponds to tremor phase likelihood of 0.1, while the inner circle corresponds to tremor phase likelihood of 0.05. (**C**) Tremor amplitude was not modulated depending on the timing of stimulation pulses with respect to the tremor cycle. Significance of tremor phase dependent tremor amplitude modulation was assessed with respect to instantaneous tremor amplitude variability when stimulation was switched off using the Wilcoxon rank sum test and significance levels corrected for multiple comparisons using the false discovery rate procedure. Circles show median change in tremor amplitude and shaded regions indicate the 95th confidence intervals of the median values.

Tremor entrainment was assessed by adapting a previously described method (Cagnan *et al.*, 2013). Tremor phase when a stimulation pulse was delivered (or when sampled at *f_T_* in DBS off condition) was divided into 20 phase bins of duration 0.3 radians. The likelihood of a tremor phase was calculated by normalizing the number of elements in each phase bin by the total number of elements. Tremor entrainment was defined as the z-score of the most likely tremor phase value, calculated by subtracting the average tremor phase likelihood off stimulation divided by the standard deviation of the tremor phase likelihood off stimulation. Effects of stimulation state on tremor entrainment were tested using a two-tailed paired Wilcoxon signed-rank test, whereas effect of stimulation site and patient group on tremor entrainment was tested using two-tailed Wilcoxon rank sum tests, as distributions were not normal (Kolmogorov-Smirnov test, *P* ≤ 0.05), and significance levels were corrected for multiple comparisons between groups using the false discovery rate procedure (Curran-Everett, 2000). For visualization purposes, tremor phase distributions were smoothed across three neighbouring phase bins; however, statistical analyses were performed on phase distributions before smoothing (Figs 1B and 2B).

### Relationship between phase and amplitude

Percentage change in instantaneous tremor amplitude was divided into 20 bins depending on the corresponding timing of the stimulation pulse with respect to the tremor cycle. Effect of stimulating at a certain tremor phase was determined by comparing tremor amplitude during stimulation to that observed in the absence of stimulation using a two-tailed Wilcoxon rank sum test (tremor amplitude distributions were not normal, Kolmogorov-Smirnov test, *P* ≤ 0.05). When DBS was switched off, instantaneous tremor amplitude and phase were sampled at *f_T_*. Significance at each phase bin was corrected for multiple comparisons using the false discovery rate procedure (Curran-Everett, 2000). For visualization purposes, phase-amplitude profiles were smoothed across three neighbouring phase bins; however, statistical analyses were performed on phase-amplitude profiles prior to smoothing (Figs 1C and 2C).

### Group phase-amplitude profile

Individual tremor phase-amplitude profiles were grouped according to DBS electrode location (i.e. subthalamic nucleus versus ventrolateral thalamus) in order to obtain the phase-amplitude profile across all patients with a given stimulation site and diagnosis. Before calculating the median profile across subjects, the tremor phase-amplitude profile from each patient was re-aligned so that 0 radians corresponded to either the tremor phase inducing maximal tremor amplification or maximal tremor suppression during stimulation at *f_T_*. We compared the effect of stimulation state at each tremor phase bin to the same bin in correspondingly realigned phase amplitude profiles derived without stimulation, using a paired Wilcoxon signed-rank test. Significance levels were corrected for multiple comparisons using the false discovery rate procedure (Curran-Everett, 2000). We opted to use a non-parametric test in order to minimize the effect of small sample size. Maximal tremor amplification and maximal tremor suppression observed during stimulation at *f_T_* were compared between the two stimulation sites and between different tremor types using the two-tailed Wilcoxon rank sum test.

### Amplitude variability during Parkinson’s disease and essential tremor

We tested how responsive tremor amplitude was to deviations in tremor frequency away from the median tremor frequency when DBS was switched off. For each patient, we sampled instantaneous tremor phase, calculated using the Hilbert transform, at the patient’s median tremor frequency and calculated the difference between two consecutive tremor phase samples. We refer to this metric as phase change. Thus phase change reflects deviations in instantaneous tremor frequency away from the median tremor frequency. As phase is a normalized measure indicating position in a tremor cycle, by keeping to phase we remove any limitations arising from tremor frequency differences between patients when performing analysis at the group level.

We divided the tremor amplitude envelope into 16 bins depending on the corresponding absolute phase change and normalized the tremor amplitude with respect to the median tremor amplitude observed at the median tremor frequency (i.e. phase change bin 0–0.2 radians). As we expected any amplitude dependency on phase to be symmetric (due to the cyclical nature of tremor) we derived histograms of amplitude change over absolute phase changes. If <70% of a given patient cohort contributed to a bin, then this bin was discarded to ensure accurate estimates of median tremor amplitude for a given phase change bin.

## Results

Eight patients with Parkinson’s disease who had been implanted with DBS electrodes into the ventrolateral thalamus, seven patients with Parkinson’s disease who had been implanted with DBS electrodes into the subthalamic nucleus and 10 patients with essential tremor who had been implanted with DBS electrodes into the ventrolateral thalamus were stimulated at the nearest integer frequency of their tremor frequency (*f_T_*). Stimulation at *f_T_* was not locked to tremor as we were not able to control the exact timing of each stimulation pulse using clinical stimulators. We instead relied on the frequency mismatch between stimulation and tremor to inform on the effects of stimulation at different parts of the tremor cycle as the stimulation and tremor drifted in and out of phase with each other (Cagnan *et al.*, 2013).

### Illustrative single subject data

The effects of DBS at *f_T_* delivered to ventrolateral thalamus are shown for a Parkinson’s disease patient with ventrolateral thalamic electrodes in Fig. 1. Median tremor frequency was 3.8 Hz when DBS was switched off (Fig. 1A: green bars). During stimulation at 4.0 Hz, median tremor frequency did not change (Fig. 1A: blue bars). If tremor phase and stimulation frequency were statistically independent, tremor phase sampled at stimulation frequency would be uniformly distributed around the unit circle (Fig. 1B). However, any statistical dependence between the two would give rise to an asymmetry in the tremor phase distribution (sampled at *f_T_*). The degree of tremor entrainment across the entire stimulation block was summarized by the standard (*z*) score of the most likely tremor phase value during stimulation with respect to tremor phase variability when DBS was turned off. The standard score of the most likely phase value during stimulation in this patient was 3.0 (Fig. 1B: blue shaded region) as opposed to 1.7 (Fig. 1B: green shaded region) when DBS was switched off. During DBS at *f_T_* the instantaneous amplitude of the tremor envelope was not significantly modulated (Fig. 1C, blue trace) with respect to instantaneous tremor amplitude variability when DBS was switched off (Fig. 1C, green trace) (two-tailed Wilcoxon rank sum test at each phase bin, degrees of freedom indicated in Supplementary Table 2).

The effects of DBS at *f_T_* delivered to the subthalamic nucleus are shown for another patient with Parkinson’s disease. The median tremor frequency was 4.6 Hz when DBS was switched off (Fig. 2A: green bars). During stimulation at 4.0 Hz, median tremor frequency did not change (Fig. 2A: blue bars). Tremor entrainment during stimulation was 4.3 (Fig. 2B: blue shaded region) as opposed to 2.5 when DBS was switched off (Fig. 2B: green shaded region). During DBS at *f_T_*, tremor amplitude was not modulated by the timing of stimulation pulses with respect to the tremor cycle (Fig. 2C, blue trace) when compared to instantaneous tremor amplitude variability when DBS was switched off (Fig. 2C, green trace) (two-tailed Wilcoxon rank sum test at each phase bin, degrees of freedom indicated in Supplementary Table 2).

The effects of stimulation of the ventrolateral thalamus at *f_T_* in a patient with essential tremor have been previously reported and, like Parkinson’s disease, showed tremor entrainment, but unlike Parkinson’s disease, demonstrated concurrent modulation of tremor amplitude depending on timing of stimulation in the tremor cycle (see Fig. 2 in Cagnan *et al.*, 2013).

### Tremor entrainment effects at the group level

**Figure 3:**
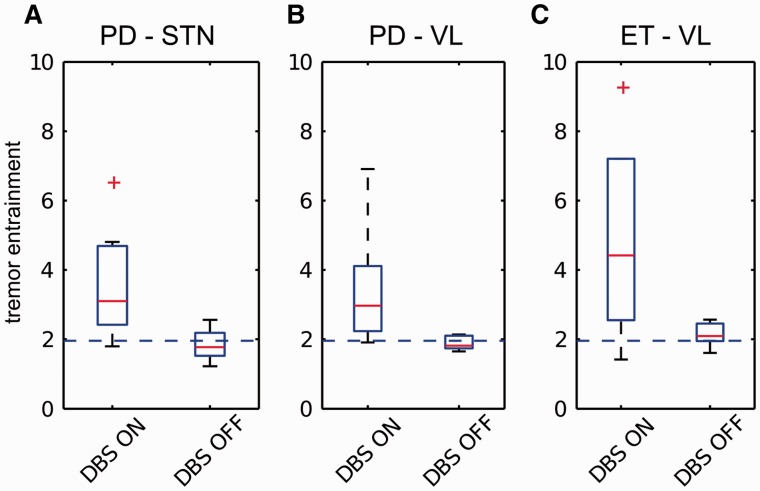
Parkinsonian and essential tremor entrainment. Tremor entrainment observed during stimulation at *f_T_* of (**A**) seven patients with Parkinson’s disease implanted in the subthalamic nucleus, (**B**) eight patients with Parkinson’s disease implanted in the ventrolateral thalamus, and (**C**) 10 patients with essential tremor implanted in the ventrolateral thalamus. Tremor entrainment during stimulation at near tremor frequencies was significantly greater than tremor entrainment observed when stimulation was switched off for both patient groups and stimulation sites. There was no difference in the level of tremor entrainment observed during stimulation at *f_T_* between the two patient groups and between the two stimulation sites. Red lines depict median values, the edges of the boxes indicate the 25th and 75th percentiles, and whiskers extend to the most extreme values observed that were not outliers. Outliers are shown as red plus symbol and defined as those values larger than *q*_75_ + 1.5 × (*q*_75_ – *q*_25_) or smaller than *q*_25_ – 1.5(*q*_75_ – *q*_25_), where *q*_25_ and *q*_75_ are the 25th and 75th percentiles, respectively. Dashed blue line depicts a z-score of 1.96. PD = Parkinson’s disease; ET = essential tremor; STN = subthalamic nucleus; VL = ventrolateral thalamus.

The spontaneous variance in tremor frequency showed only a weak trend towards being greater in parkinsonian rest tremor than essential tremor (*P* = 0.0963, df = 23, two sided Student’s *t*-test). As summarized in Fig. 3 and Table 3, all three patient groups demonstrated significant entrainment of tremor during stimulation at *f_T_* compared to when stimulation was switched off. Tremor entrainment during stimulation at *f_T_* did not differ between patient groups. Note that there were no significant differences in stimulation parameters between groups (Tables 1 and 2).

**Table 3:** Tremor entrainment

	PD-STN	PD-VL	ET-VL
% Patients showing entrainment	85% (*n* = 7)	75% (*n* = 8)	80% (*n* = 10)
Median entrainment score and range during stimulation at *f_T_*	3.0 (1.8–10.8)	2.7 (1.9–6.9)	4.4 (1.4–27)
Median entrainment score and range unstimulated (sampled at *f_T_*)	1.7 (1.2–2.3)	1.8 (1.6–2.1)	2.0 (1.6–2.5)
Difference between stimulated and unstimulated entrainment scores	*P* = 0.031	*P* = 0.008	*P* = 0.014
Group differences in entrainment during stimulation	PD-STN versus PD-VL	PD-STN versus ET-VL	PD-VL versus ET-VL
	*P* = 0.779	*P* = 0.6	*P* = 0.274

All comparisons used two-tailed paired Wilcoxon signed-rank tests for within group comparisons (df PD-STN: seven subjects; df PD-VL: eight subjects; df ET-VL: 10 subjects) and two-tailed Wilcoxon rank sum tests for across group comparisons (PD-STN: seven subjects; PD-VL: eight subjects; ET-VL: 10 subjects).

PD = Parkinson’s disease; ET = essential tremor; STN = subthalamic nucleus; VL = ventrolateral thalamus.

### Tremor amplitude effects at the group level

**Figure 4:**
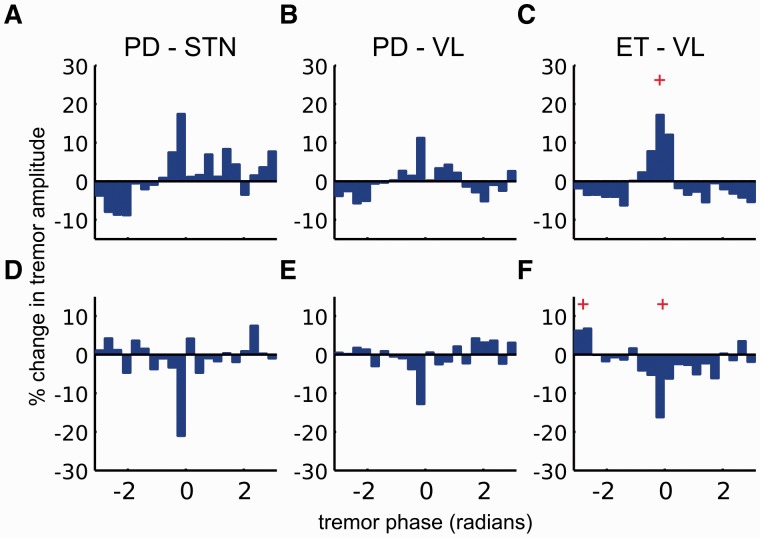
Group phase-amplitude profiles for parkinsonian and essential tremor. Median tremor phase-amplitude profile of the seven patients with Parkinson’s disease with subthalamic DBS electrodes during stimulation at *f_T_* is shown when individual phase amplitude profiles were aligned to (**A**) maximal tremor amplification, (**D**) maximal tremor suppression. Similarly in (**B**) and (**E**), median tremor phase amplitude profiles of the eight patients with Parkinson’s disease implanted with thalamic DBS electrodes are shown following alignment to maximal tremor amplification and maximal tremor suppression, respectively. None of the amplitude changes observed during stimulation were significantly different from tremor amplitude variability observed when DBS was switched off (two-tailed paired Wilcoxon signed-rank test performed at each tremor phase bin corrected for multiple comparisons across 20 tremor phase bins using the false discovery rate procedure). However, median tremor phase amplitude profiles of the 10 essential tremor patients with thalamic electrodes when either aligned to (**C**) maximal amplification or (**F**) maximal suppression show significant differences in tremor amplitude when compared to tremor amplitude variability observed when DBS was switched off (two-tailed paired Wilcoxon signed-rank test). Significance is indicated with a red plus symbol. PD = Parkinson’s disease; ET = essential tremor; STN = subthalamic nucleus; VL = ventrolateral thalamus.

We also investigated whether stimulation timing with respect to the tremor cycle gave rise to any changes in the instantaneous amplitude of the tremor envelope at the group level. The relationship between tremor phase and instantaneous tremor amplitude need not be the same across all patients, as this will vary according to the precise constellation of muscles and muscle forces involved in the orchestration of the tremor. Accordingly we aligned individual phase-amplitude profiles so that 0 radians corresponded to either (i) maximal tremor amplification (Fig. 4A–C); or (ii) maximal tremor suppression (Fig. 4D–F) before calculating the median profile across patients with a given stimulation site and diagnosis. In patients with Parkinson’s disease, tremor amplitude was not significantly modulated by stimulation at *f_T_* regardless of stimulation site and the part of the tremor cycle the stimulation pulse landed at [two-tailed paired Wilcoxon sign-rank test at each phase bin: df = 7 (Fig. 4A and D); df = 8 (Fig. 4B and E)]. Moreover, there was no difference in either maximal tremor amplification or suppression between stimulation of the two sites in patients with Parkinson’s disease (amplification, *P* = 0.189 and suppression, *P* = 0.054; number of Parkinson’s disease patients with subthalamic implants = 7, with ventrolateral implants = 8; two-tailed Wilcoxon rank sum tests).

These findings in Parkinson’s disease were in stark contrast with the tremor amplitude modulation observed in patients with essential tremor, where significant tremor amplification and suppression were present relative to when DBS was switched off [two-tailed paired Wilcoxon sign-rank test at each phase bin: df = 10 (Fig. 4C and F)]. This remained significant when contrasted with the maximal tremor amplification in the Parkinson’s disease group with thalamic DBS electrodes (tremor amplification *P* = 0.0343, number of Parkinson’s disease patients with ventrolateral implants = 8, number of essential tremor patients with ventrolateral implants = 10; two-tailed Wilcoxon rank sum tests). Maximal tremor suppression did not differ between the two patient groups stimulated in the thalamus (tremor suppression, *P* = 0.1457).

### Why are tremor amplitude effects found in essential tremor but not Parkinson’s disease?

**Figure 5:**
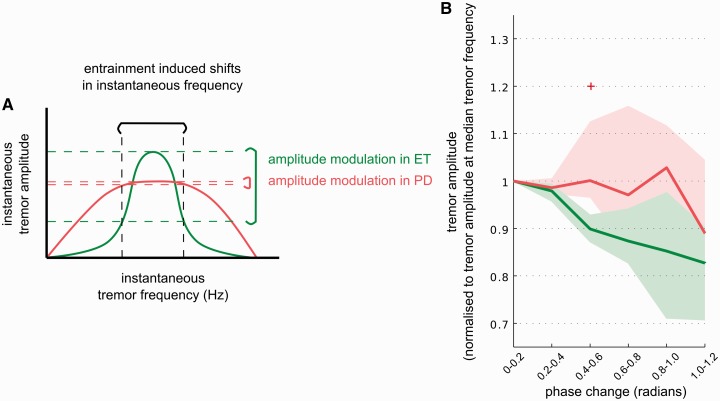
Tremor amplitude variability during Parkinson’s disease and essential tremor. (**A**) Schematic of resonance functions of the oscillators or systems of linked oscillators underlying Parkinson’s disease (PD) and essential tremor (ET). Parkinsonian tremor is shown in red with a platykurtic resonance function. Hence changes in instantaneous frequency caused by extrinsic driving forces lead to a relatively small change in instantaneous amplitude, measured from the tremor envelope. Essential tremor is shown in green with a more leptokurtic resonance function. Thus changes in instantaneous frequency caused by extrinsic driving lead to a relatively big change in instantaneous tremor amplitude. (**B**) Group data for change in tremor amplitude for a certain change in absolute phase. Only phase changes to which at least 70% of the patient cohort contributed for a given pathology are shown. Serial independent Mann Whitney tests at each phase change bin indicate that Parkinson’s and essential tremor response to phase changes differ (*P* = 0.007 at phase change bin 0.4–0.6 radians). Significance is indicated with a red plus symbol.

The above results in Parkinson’s disease present a paradox. Stimulation at *f_T_* could entrain tremor but made little or no difference to tremor amplitude. This implies that the underlying oscillator or system of linked oscillators driving tremor has a platykurtic (relatively flat) resonance function (see red curve in the schematic in Fig. 5A). Hence changes in instantaneous frequency caused by extrinsic driving forces (e.g. deep brain stimulation (DBS) at *f_T_*) led to a small change in the instantaneous amplitude of the tremor envelope. In contrast, in essential tremor we found both tremor entrainment and amplitude effects, consistent with an underlying oscillator or system of linked oscillators that has a more leptokurtic (relatively peaked) resonance function (see green curve in Fig. 5A). Thus changes in instantaneous frequency caused by extrinsic driving lead to a relatively big change in the instantaneous amplitude of the tremor envelope.

We examined the effects of spontaneous variations in the instantaneous tremor frequency on parkinsonian rest tremor and essential tremor when DBS was switched off. Examples of such data from a case with Parkinson’s disease and another with essential tremor are shown in Supplementary Fig. 1. To analyse this at the group level, we sampled tremor phase at median tremor frequency and took the difference in radians, i.e. phase change, between consecutive phase samples. This metric characterizes how stable tremor frequency is around the median tremor frequency. Tremor with abrupt frequency changes would have large phase changes whereas tremor with a relatively stationary frequency would have small phase changes. For each patient, we evaluated change in the tremor amplitude for a certain phase change. Tremor amplitude at each phase change bin was normalized with respect to the median tremor amplitude observed when there was no phase change (i.e. phase change bin 0–0.2 radians). Figure 5B shows change in tremor amplitude for a given absolute phase change across all patients with Parkinson’s disease regardless of stimulation site (*n* = 15) when DBS was not applied. Tremor amplitude barely changed with abrupt changes in tremor frequency over the range with sufficient data to analyse. In contrast, in the essential tremor patient cohort (*n* = 10) tremor amplitude was much more reactive to changes in instantaneous tremor frequency, and the instantaneous amplitude of the tremor envelope was only relatively fixed at frequencies near to the median tremor frequency.

#### Consecutive stimuli at phase values favouring amplification or suppression

Figures 1 and 2 highlight tremor amplitude dependency on tremor phase, without taking into account the effect of previous stimulation pulses on tremor amplitude. To test whether stimulation history had any cumulative effect on tremor amplitude, percentage changes in instantaneous tremor amplitude were grouped into bins based on whether the corresponding tremor phase promoted tremor suppression or amplification, and depending on how many preceding stimulation pulses landed on tremor phases promoting the same type of amplitude change. Repeated measures ANOVA revealed that there was no cumulative amplitude effect of the number of consecutive stimuli applied at tremor amplification promoting parts of the tremor cycle and no effect of stimulation state (i.e. stimulation at *f_T_* versus stimulation off) at either stimulation site (Supplementary material). The same held for tremor suppression (Supplementary material) in patients with Parkinson’s disease. In contrast, similar analysis revealed significant cumulative effects of increasing numbers of consecutive stimuli applied at tremor suppression promoting parts of the tremor cycle during stimulation of the ventrolateral thalamus in essential tremor (Cagnan *et al.*, 2013).

## Discussion

We have shown that both thalamic and subthalamic stimulation at *f_T_* alters the temporal profile of parkinsonian rest tremor and significantly entrains tremor. Critically, though, there was no significant stimulation timing-dependent change in instantaneous tremor amplitude when compared to tremor amplitude variability observed without stimulation. This is in stark contrast to essential tremor where thalamic DBS at *f_T_* both entrained tremor and modulated instantaneous tremor amplitude depending on the tremor phase at which stimulation was applied. Parkinsonian rest and essential tremor also differed in the relative tolerance of their amplitudes to spontaneous changes in instantaneous tremor frequency, with the amplitude of parkinsonian rest tremor demonstrating greater tolerance to spontaneous changes in instantaneous tremor frequency than in essential tremor. Thus the oscillators or system of linked oscillators underlying Parkinson’s rest tremor and essential tremor have relatively platykurtic and leptokurtic resonance functions, respectively. This intrinsic property of rest tremor in Parkinson’s disease explains why instantaneous tremor amplitude did not change during stimulation at *f_T_,* despite the inevitable changes in instantaneous frequency induced in the process of entrainment to a slightly different frequency. In contrast, the leptokurtic resonance function in essential tremor meant that the changes in instantaneous frequency induced in the process of entrainment to a slightly different frequency were accompanied by changes in instantaneous tremor amplitude.

We hypothesize that platykurtic and leptokurtic tremor resonance functions reflect the nature of the neural circuits underpinning the different tremor types, while allowing for these centrally determined resonance functions to be further modified by the resonance characteristics of the tremulous limb. So how important might changes in the stiffness (*k*) or inertia (*I*) of the limb be in explaining the shape of the tremor resonance functions in parkinsonian rest and essential tremor, given that the two data sets were collected with the limb in different positions? Here EMG recordings might have been helpful, as significant peripheral factors would be expected to lead to dissociation between tremor resonance functions determined by EMG and accelerometry, as the latter also reflects purely mechanical factors. Such a dissociation should be most obvious as a shift in tremor frequency, ω, as dictated by $\omega =\sqrt{k/I}$, but even frank weighting of limbs makes little difference to tremor frequency in these two conditions (Hallett, 1998). Similarly, tremor frequency did not differ significantly between our patient groups, despite the different disease-specific tremor recording conditions. Another observation that would suggest a relatively limited contribution from peripheral effects is that the tremor resonance function of Parkinson’s disease rest tremor did not differ between dopamine responsive (implanted in the subthalamic nucleus) and relatively dopamine unresponsive (implanted in the ventrolateral thalamus) groups even though the former would, through patient selection, have been expected to have greater limb stiffness.

On the other hand, peripheral factors related to the rest-posture difference could not have accounted for the lack of cumulative effects of increasing numbers of consecutive stimuli applied at tremor suppression or amplification promoting parts of the tremor cycle in Parkinson’s disease, as opposed to essential tremor (Supplementary Figs 2 and 3). The ability of stimulation to entrain tremor also suggests that central factors are critical in determining tremor dynamics. In Parkinson’s disease the entrainment of tremor through stimulation in the ventrolateral thalamus and subthalamic nucleus strongly implies that both regions are involved in the tremor pacemaker circuit of rest tremor (Hansel *et al.*, 1995; Ermentrout, 1996; Smeal *et al.*, 2010). The involvement of ventrolateral thalamus and subthalamic nucleus in the Parkinson’s disease tremor pacemaker circuit complements the clinical observation that high frequency stimulation of the subthalamic nucleus may be as effective as high frequency stimulation of the ventrolateral thalamus in suppressing Parkinson’s disease tremor (Krack *et al.*, 1998), although tremor suppression need not necessarily demonstrate interference with a pacemaker circuit, and could potentially implicate interference with the pacemaker circuit’s outflow or mechanisms amplifying this outflow.

### Implications for theories of rest tremor circuitry in Parkinson’s disease

There are several hypotheses regarding the mechanism underlying rest tremor generation in Parkinson’s disease. The first identifies thalamus as a key nucleus in the tremor pacemaker circuit (Llinás, 1984, 1988). The basis for this hypothesis lies in the ion channel properties of thalamocortical relay neurons, which enable these neurons to generate oscillations at around tremor frequency (Llinás, 1988; Llinás *et al.*, 2005). Oscillations at tremor frequency can be triggered by modulation of thalamic excitability through hyperpolarization or by reduction in excitatory drive (Llinás *et al.*, 2005). In Parkinson’s disease, depletion of dopamine increases the firing rate of globus pallidus internus neurons (Filion and Tremblay, 1991), resulting in a net increase in hyperpolarization of pallidal input-receiving neurons in the thalamus (Albin *et al.*, 1989). Dopaminergic medication may reverse this and thereby ameliorate rest tremor (Jankovic and Tolosa, 2007). However, this theory affords only a permissive role to basal ganglia output, while the tremor entrainment observed during stimulation of the subthalamic nucleus implies that the latter nucleus is also a core part of the tremor pacemaker circuit. Neither does it explain why high frequency stimulation of the subthalamic nucleus, which has been shown to increase the firing rate of globus pallidus internus neurons, suppresses tremor, because this increase in firing rate would imply an increase in the net inhibition of the thalamus (Hashimoto *et al.*, 2003). An alternative interpretation of the thalamic pacemaker theory is that it is the reduction in excitatory input from the cerebellum that gives rise to thalamic oscillations at tremor frequencies. This interpretation is supported by tremor-related discharges that are also found in the cerebellar input receiving portions of the ventrolateral thalamus (Magnin *et al.*, 2000), MEG studies that demonstrate a tremor-related oscillatory network involving a cerebello-diencephalic-cortical loop (Timmermann *et al.*, 2002), and suppression of tremor due to surgery intended to lesion or stimulate cerebellar input zones in the thalamus (Benabid *et al.*, 1991; Lenz *et al.*, 1995). This, however, would also fail to explain the tremor entrainment during stimulation of the subthalamic nucleus unless this activated cerebello-thalamic fibres passing near the subthalamic nucleus. Activation of these fibres has been suggested to underlie the clinical efficacy of subthalamic DBS on the basis of very short-latency potentials observed in cerebellar input receiving regions of the thalamus during subthalamic DBS in non-human primates made parkinsonian with the neurotoxin 1-methyl-4-phenyl-1,2,3,6-tetrahydropyridine (Xu *et al.*, 2008). Yet an effect on tremor through this mechanism would not account for tremor-related neuronal activity in the subthalamic nucleus itself (Rodriguez-Oroz *et al.*, 2001), nor the coherence between subthalamic local field potential and tremor EMG (Hirschmann *et al.*, 2013).

Another hypothetical schema ascribes a key role to the recurrent loop between the external part of the globus pallidus and the subthalamic nucleus, and posits that this loop can generate synchronized oscillations following excitability and connection strength changes triggered by dopamine depletion (Terman *et al.*, 2002). This theory is supported by the observation that neuronal discharges in the subthalamic nucleus and globus pallidus are correlated with rest tremor bursts in patients with Parkinson’s disease or in non-human primates made parkinsonian with the neurotoxin 1-methyl-4-phenyl-1,2,3,6-tetrahydropyridine (Hutchison *et al.*, 1997; Bergman *et al.*, 1998; Hurtado *et al.*, 1999; Rodriguez-Oroz *et al.*, 2001; Heimer *et al.*, 2006). Additionally, the effectiveness of subthalamic DBS in tremor suppression further supports the importance of the basal ganglia in Parkinson’s disease rest tremor (Fasano *et al.*, 2012). However, this theory does not explain the data showing tremor-related firing patterns in the cerebellar input receiving parts of the thalamus (Lenz *et al.*, 1994; Magnin *et al.*, 2000).

A further hypothesis, the dimmer-switch hypothesis, has recently been proposed by Helmich *et al.* (2012, 2013). This suggests that the basal ganglia can trigger tremor episodes while the cerebello-thalamo-cortical circuit modulates tremor amplitude. This hypothesis is based on data that show increased cerebral activity in the basal ganglia and cerebello-thalamo-cortical circuit in which only the latter exhibits activity changes related to slow modulations in tremor amplitude (Helmich *et al.*, 2011). Although this hypothesis is attractive in that it integrates evidence linking both basal ganglia and thalamic networks to Parkinson’s disease tremor and might explain why high frequency stimulation of both the subthalamic nucleus and of the ventrolateral thalamus can be effective in controlling Parkinson’s disease tremor, our results implicate both the subthalamic nucleus and thalamus in tremor pace-making. Moreover, despite tremor entrainment, we did not observe significant modulation of the instantaneous amplitude of the tremor envelope from either site in excess of resting tremor variability, arguing that any tremor amplitude modulation possibly occurs beyond these sites. Interestingly, stimulation of the motor cortex is reported to both entrain and modulate the amplitude of Parkinson’s disease rest tremor depending on the tremor phase at which stimulation is applied (Brittain *et al.*, 2013). The latter study used different stimulation and analytic techniques but raises the possibility that motor cortex is not only involved in an extended tremor pacemaker circuit, but that amplitude modulation could potentially be exerted at the level of the motor cortex or its outflow (see also Hirschmann *et al.*, 2013). This would also be consistent with the cortico-muscular coherence at tremor frequency and its first harmonic (Volkmann *et al.*, 1996; Hellwig *et al.*, 2000).

### Contrasts with essential tremor

The effects of stimulation at *f_T_* in our patients with Parkinson’s disease were strikingly different to those in patients with essential tremor even when consideration was limited to those patients in each group with electrodes in the ventrolateral thalamus (Cagnan *et al.*, 2013). Stimulation in both conditions afforded similar levels of tremor entrainment, so that stimulation efficacy was comparable by this measure, and indeed stimulation voltages, frequencies and pulse durations were similar in the two patient cohorts. However only low frequency stimulation of patients with essential tremor was able to modulate (suppress or amplify) tremor amplitude depending on the timing of stimulation pulses with respect to tremor phase beyond the natural variability in postural tremor. This points to differences in the resonance characteristics of the underlying oscillator circuits in Parkinson’s disease and essential tremor, and this was further corroborated by the analysis of amplitude dependency on spontaneous changes in instantaneous frequency when DBS was not applied.

Patients with Parkinson’s disease differed from those with essential tremor during stimulation of the ventrolateral thalamus also in terms of the relative lack of cumulative effects with consecutive stimuli delivered at tremor phases that promoted tremor suppression or amplification (Cagnan *et al.*, 2013). Even selecting those short runs of stimuli that were consecutively delivered at similar tremor phases failed to recover a significant amplitude effect in Parkinson’s disease. This is important, as it makes the weak trend towards increased frequency variability of Parkinson’s disease rest tremor compared to essential tremor a less likely reason for the relatively weaker phase-dependent amplitude modulation in the former. Reports are mixed within the literature as to whether tremor amplitude variance is greater in parkinsonian rest tremor than in essential tremor (Gao, 2004) or not (O’Suilleabhain and Matsumoto, 1998; Jankovic and Tolosa, 2007).

Together, the above differences suggest that the circuitry underpinning parkinsonian rest tremor and essential tremor differs in its functional characteristics at the level of the ventrolateral thalamus and its connections. Given that the tremor frequencies observed in the two pathologies overlap and tremor frequency variance is the same in the two pathologies (O'Suilleabhain and Matsumoto, 1998; Jankovic and Tolosa, 2007), the circuitry sustaining essential tremor must be more strongly coupled and prescriptive in the relative timing of neural activity between elements, necessary for sustaining essential tremor. Thus perturbation by phase advancing or slowing of one (or more) element(s) through tremor frequency matched stimulation can disrupt the recurrent loop, perhaps as presynaptic spiking activity now falls in refractory periods of postsynaptic neural activity, and diminish tremor amplitude. The corollary of this is that tremor amplitude in essential tremor is more sensitive to spontaneous variability in instantaneous tremor frequency than in Parkinson’s disease. In contrast, the circuitry underpinning parkinsonian rest tremor appears to be weakly coupled and more forgiving in the relative timing between elements necessary for sustaining neural activity that drives rest tremor. Phase advancing or slowing of one element had less of an effect on tremor amplitude (and, possibly less of an effect on spike timing dependent plasticity, if this underlies cumulative effects). Whether the difference between the two pathologies is innate or due to dynamic changes in circuit state with posture, however, remains to be explored (Brittain and Brown, 2013).

It is important to consider whether demographic, clinical or methodological differences could contribute to the different tremor characteristics between the two groups. The only significant demographic and clinical difference between the essential tremor and parkinsonian patients implanted in the ventrolateral thalamus in this small cohort were the shorter disease duration and the use of dopaminergic medications in Parkinson’s disease. The influence of these features in determining tremor entrainment is unclear, but several factors suggest that medication may not have played a significant role in determining group differences. Tremor-dominant Parkinson’s disease patients implanted with depth electrodes in ventrolateral thalamus were poorly dopaminergic responsive. This makes it less likely that any treatment with dopaminergic medication in this group accounted for differences in tremor features with respect to essential tremor. Moreover, tremor entrainment and resonance functions were similar for both dopamine responsive Parkinson’s disease patients implanted in the subthalamic nucleus and relatively dopamine unresponsive patients implanted in the ventrolateral thalamus. This further suggests that medication as such had little effect.

The narrower frequency-amplitude tolerance in essential tremor could arise from finer tuning of the central drive or its outflow to the periphery with posture. This may help explain the stimulation timing dependent change in tremor amplitude in this condition as opposed to Parkinsonian rest tremor. However, in the latter case we should also entertain the possibility that resting tremor amplitude is determined at a downstream site, and the subthalamic nucleus and ventrolateral thalamus are solely involved in pacing tremor and not determining its amplitude. Motor cortex is one possible region where resting tremor amplitude could be determined. This is supported by significant amplitude modulation of parkinsonian resting tremor (tremor severity quantified with accelerometry as here) with transcranial alternating current stimulation of the motor cortex (Brittain *et al.*, 2013).

### Clinical relevance and conclusion

The more forgiving nature of the Parkinson’s disease rest tremor circuit with respect to phase shifts in its components has significant implications with respect to how susceptible parkinsonian tremor will be to phase interference stimulation techniques under development. These are techniques that seek to interact with oscillators at the key phases that either promote instantaneous suppression or, over many cycles, change synaptic weights through plasticity (Tass and Majtanik, 2006; Tass *et al.*, 2012; Cagnan *et al.*, 2013). While the definitive test of the use of these phase interference techniques in Parkinson’s disease awaits the tracking of tremor phase and the delivery of stimuli at the optimal phase for tremor suppression over more prolonged periods lest this harnesses even weak cumulative effects in Parkinson’s disease, our results suggest that these techniques might be better piloted and refined in essential tremor. The prescriptive nature of essential tremor pathophysiology suggests that accurately timed stimulation pulses could be effective in suppressing tremor amplitude in this condition, either by decoupling the neural circuit driving tremor and/or by entraining the pacemaker circuit so that there are instantaneous frequency changes that lie outside of the narrow frequency-amplitude tolerance range in this condition. However, in Parkinson’s disease, the broad frequency-amplitude tolerance of the underlying tremor circuit suggests that its median frequency will have to be entrained to frequencies outside of this broad tolerance zone, before stimuli applied systematically at certain tremor phases can induce either tremor amplitude potentiation or, as desired clinically, amplitude suppression.

Our results also suggest that both the subthalamic nucleus and ventrolateral thalamus are involved in the tremor-pacemaker circuit of Parkinson’s disease, as evinced by the ability of stimulation at *f_T_* at either site to entrain rest tremor. The clinical effects of high frequency stimulation of the two targets on parkinsonian tremor would further suggest that the two sites are essential components of the tremor circuit (Benabid *et al.*, 1991; Krack *et al.*, 1998). Nevertheless, we are still left with the paradox that the intended surgical target in stimulation of the ventrolateral thalamus is, at least in principle, the cerebellar receiving and not the basal ganglia receiving zone (Benabid *et al.*, 1991; Lenz *et al.*, 1994). This leaves us to speculate that the interaction between the two systems in Parkinson’s disease implied by our results occurs either at the level of cerebral motor cortical areas (Percheron *et al.*, 1996), or, as recently highlighted, through the di-synaptic connections from the subthalamic nucleus to the cerebellar cortex (Bostan *et al.*, 2010) and from the dentate nucleus of the cerebellum back to the striatum (Hoshi *et al.*, 2005).

## Funding

This work was funded by the Medical Research Council and the National Institute of Health Research, Oxford Biomedical Research Centre. Some of the work was supported by the NIHR Oxford cognitive health Clinical Research Facility, Oxford, and some work was undertaken at UCL/UCLH, which is partly funded by the Department of Health NIHR Biomedical Research Centres funding scheme. The Functional Neurosurgery unit, UCL Institute of Neurology, is supported by the Parkinson's Appeal and the Sainsbury Monument Trust.

## Supplementary material

Supplementary material is available at *Brain* online.

## Abbreviation

DBS: deep brain stimulation

## References

[awu250-B1] Albin RL, Young AB, Penney JB (1989). The functional anatomy of basal ganglia disorders. Trends Neurosci.

[awu250-B2] Bain PG, Findley LJ, Atchison P, Behari M, Vidailhet M, Gresty M (1993). Assessing tremor severity. J Neurol Neurosurg Psychiatry.

[awu250-B3] Benabid AL, Pollak P, Gervason C, Hoffmann D, Gao DM, Hommel M (1991). Long-term suppression of tremor by chronic stimulation of the ventral intermediate thalamic nucleus. Lancet.

[awu250-B4] Bergman H, Feingold A, Nini A, Raz A, Slovin H, Abeles M (1998). Physiological aspects of information processing in the basal ganglia of normal and parkinsonian primates. Trends Neurosci.

[awu250-B5] Bostan AC, Dum RP, Strick PL (2010). The basal ganglia communicate with the cerebellum. Proc Natl Acad Sci USA.

[awu250-B6] Brittain J-S, Brown P (2013). The many roads to tremor. Exp Neurol.

[awu250-B7] Brittain J-S, Probert-Smith P, Aziz TZ, Brown P (2013). Tremor suppression by rhythmic transcranial current stimulation. Curr Biol.

[awu250-B8] Cagnan H, Brittain J-S, Little S, Foltynie T, Limousin P, Zrinzo L (2013). Phase dependent modulation of tremor amplitude in essential tremor through thalamic stimulation. Brain.

[awu250-B9] Curran-Everett D (2000). Multiple comparisons: philosophies and illustrations. Am J Physiol Regul Integr Comp Physiol.

[awu250-B10] Ermentrout B (1996). Type I membranes, phase resetting curves, and synchrony. Neural Comput.

[awu250-B11] Fasano A, Daniele A, Albanese A (2012). Treatment of motor and non-motor features of Parkinson’s disease with deep brain stimulation. Lancet Neurol.

[awu250-B12] Filion M, Tremblay L (1991). Abnormal spontaneous activity of globus pallidus neurons in monkeys with MPTP-induced parkinsonism. Brain Res.

[awu250-B13] Gao PJB (2004). Analysis of amplitude and frequency variations of essential and Parkinsonian tremors. Med Biol Eng Comput.

[awu250-B14] Hallett M (1998). Overview of human tremor physiology. Mov Disord.

[awu250-B15] Hansel D, Mato G, Meunier C (1995). Synchrony in excitatory neural networks. Neural Comput.

[awu250-B16] Hashimoto T, Elder CM, Okun MS, Patrick SK, Vitek JL (2003). Stimulation of the subthalamic nucleus changes the firing pattern of pallidal neurons. J Neurosci.

[awu250-B17] Heimer G, Rivlin-Etzion M, Bar-Gad I, Goldberg JA, Haber SN, Bergman H (2006). Dopamine replacement therapy does not restore the full spectrum of normal pallidal activity in the 1-methyl-4-phenyl-1,2,3,6-tetra-hydropyridine primate model of Parkinsonism. J Neurosci.

[awu250-B18] Hellwig B, Häussler S, Lauk M, Guschlbauer B, Köster B, Kristeva-Feige R (2000). Tremor-correlated cortical activity detected by electroencephalography. Clin Neurophysiol.

[awu250-B19] Helmich RC, Hallett M, Deuschl G, Toni I, Bloem BR (2012). Cerebral causes and consequences of parkinsonian resting tremor: a tale of two circuits?. Brain.

[awu250-B20] Helmich RC, Janssen MJR, Oyen WJG, Bloem BR, Toni I (2011). Pallidal dysfunction drives a cerebellothalamic circuit into Parkinson tremor. Ann Neurol.

[awu250-B21] Helmich RC, Toni I, Deuschl G, Bloem BR (2013). The pathophysiology of essential tremor and Parkinson’s tremor. Curr Neurol Neurosci Rep.

[awu250-B22] Hirschmann J, Hartmann CJ, Butz M, Hoogenboom N, Özkurt TE, Elben S (2013). A direct relationship between oscillatory subthalamic nucleus–cortex coupling and rest tremor in Parkinson’s disease. Brain.

[awu250-B23] Hoshi E, Tremblay L, Féger J, Carras PL, Strick PL (2005). The cerebellum communicates with the basal ganglia. Nat Neurosci.

[awu250-B24] Hua SE, Lenz FA (2005). Posture-related oscillations in human cerebellar thalamus in essential tremor are enabled by voluntary motor circuits. J Neurophysiol.

[awu250-B25] Hurtado JM, Gray CM, Tamas LB, Sigvardt KA (1999). Dynamics of tremor-related oscillations in the human globus pallidus: a single case study. Proc Natl Acad Sci USA.

[awu250-B26] Hutchison WD, Lozano AM, Tasker RR, Lang AE, Dostrovsky JO (1997). Identification and characterization of neurons with tremor-frequency activity in human globus pallidus. Exp Brain Res.

[awu250-B27] Jankovic J, Tolosa E

[awu250-B28] Krack P, Benazzouz A, Pollak P, Limousin P, Piallat B, Hoffmann D (1998). Treatment of tremor in Parkinson’s disease by subthalamic nucleus stimulation. Mov Disord.

[awu250-B29] Lenz FA, Kwan HC, Martin RL, Tasker RR, Dostrovsky JO, Lenz YE (1994). Single unit analysis of the human ventral thalamic nuclear group Tremor-related activity in functionally identified cells. Brain.

[awu250-B46] Lenz FA, Normand SL, Kwan HC, Andrews D, Rowland LH, Jones MW (1995). Statistical prediction of the optimal site for thalamotomy in parkinsonian tremor. Mov. Disord.

[awu250-B30] Llinás R (1984). Rebound excitation as the physiological basis for tremor: a biophysical study of the oscillatory properties of mammalian central neurons *in vitro*. Movement disorders: tremor.

[awu250-B31] Llinás RR (1988). The intrinsic electrophysiological properties of mammalian neurons: insights into central nervous system function. Science.

[awu250-B32] Llinás R, Urbano FJ, Leznik E, Ramírez RR, van Marle HJF (2005). Rhythmic and dysrhythmic thalamocortical dynamics: GABA systems and the edge effect. Trends Neurosci.

[awu250-B33] Magnin M, Morel A, Jeanmonod D (2000). Single-unit analysis of the pallidum, thalamus and subthalamic nucleus in parkinsonian patients. Neuroscience.

[awu250-B34] Marple SL

[awu250-B35] O’Suilleabhain PE, Matsumoto JY (1998). Time-frequency analysis of tremors. Brain J Neurol.

[awu250-B47] Oliver L (1949). Surgery in Parkinson’s disease division of lateral pyramidal tract for tremor: report on forty-eight operations. The Lancet.

[awu250-B36] Percheron G, François C, Talbi B, Yelnik J, Fénelon G (1996). The primate motor thalamus. Brain Res Rev.

[awu250-B37] Reck C, Florin E, Wojtecki L, Krause H, Groiss S, Voges J (2009). Characterisation of tremor-associated local field potentials in the subthalamic nucleus in Parkinson’s disease. Eur J Neurosci.

[awu250-B38] Rodriguez-Oroz MC, Rodriguez M, Guridi J, Mewes K, Chockkman V, Vitek J (2001). The subthalamic nucleus in Parkinson’s disease: somatotopic organization and physiological characteristics. Brain.

[awu250-B39] Smeal RM, Ermentrout GB, White JA (2010). Phase-response curves and synchronized neural networks. Philos Trans R Soc Lond B Biol Sci.

[awu250-B40] Tass PA, Majtanik M (2006). Long-term anti-kindling effects of desynchronizing brain stimulation: a theoretical study. Biol Cybern.

[awu250-B41] Tass PA, Qin L, Hauptmann C, Dovero S, Bezard E, Boraud T (2012). Coordinated reset has sustained aftereffects in Parkinsonian monkeys. Ann Neurol.

[awu250-B42] Terman D, Rubin JE, Yew AC, Wilson CJ (2002). Activity Patterns in a model for the subthalamopallidal network of the basal Ganglia. J Neurosci.

[awu250-B43] Timmermann L, Gross J, Dirks M, Volkmann J, Freund H-J, Schnitzler A (2002). The cerebral oscillatory network of parkinsonian resting tremor. Brain.

[awu250-B44] Volkmann J, Joliot M, Mogilner A, Ioannides AA, Lado F, Fazzini E (1996). Central motor loop oscillations in parkinsonian resting tremor revealed magnetoencephalography. Neurology.

[awu250-B48] Walker AE, Schaltenbrand G, Walker AE (1982). Stereotaxic surgery for tremor. In: Stereotaxy for the human brain: anatomical Physiological and clinical applications. Thieme, Stuttgart.

[awu250-B45] Xu W, Russo GS, Hashimoto T, Zhang J, Vitek JL (2008). Subthalamic nucleus stimulation modulates thalamic neuronal activity. J Neurosci.

